# Fungal contaminants in Turkish bottled water and their mycotoxin-producing potential

**DOI:** 10.3205/dgkh000570

**Published:** 2025-07-14

**Authors:** Gülhan Tunç, Fusun Uçar, Osman Telli, Nelson Lima, Oana-Alina Boiu-Sicuia

**Affiliations:** 1 Ege University, Department of Biology, Laboratory of Basic and Industrial Microbiology, Izmir, Turkey; 2 Kırklareli University, Department of Molecular Biology and Genetics, Kırklareli, Turkey; 3 Universidade do Minho, Environmental Biotechnology and Bioengineering, Laboratory of Applied Mycology, Braga, Portugal; 4 University of Agronomic Sciences and Veterinary Medicine of Bucharest, Faculty of Biotechnologies, Bucharest, Romania; 5 Research – Development Institute for Plant Protection, Bucharest, Romania

**Keywords:** bottled water, fungal contaminants, Cladosporium, Aspergillus, Penicillium, alternaria, mycotoxin-producing potential

## Abstract

**Introduction:**

The chemical composition and microbial contaminants of commercially bottled water are managed according to public health regulations, but there are gaps in these criteria for fungal agents. For this reason, commercial bottled water brands filled in 19-L polycarbonate bottles from various water sources in western Anatolia and sold in Izmir and neighboring provinces were randomly selected and analyzed.

**Materials and methods:**

Fungal screening was carried out in water samples using the membrane filtration technique. The number of fungi was counted in colony-forming units (cfu)/100 ml. All fungi isolated from packaged water samples were initially phenotypically analyzed for initial preliminary identification. Amplification of the well-conserved internal transcribed spacer (ITS) region was performed by polymerase chain reaction (PCR) using internal transcribed spacer (ITS)1/ITS4 primers for genotypic identification of the isolates. Using the basic local alignment search tool (BLAST), sequencing results were compared with the National Center for Biotechnology Information (NCBI) database, and species assignments were made for 31 samples. 25 moulds and 6 yeasts were identified, and molecular identification was supported by the actin gene region.

The mycotoxin formation potential of the isolated Penicillium, Aspergillus, Alternaria and *Cladosporium* spp. was also screened using the ammonium vapor test, LC-MS and HPLC. The amount of citrinin, the metabolite produced by the *Penicillium citrinum* B4 strain, was determined by liquid chromatography-mass spectrometry.

**Results:**

The most dominant strains detected in the analysis of water samples were Cladosporium, Penicillium and Alternaria. With 411.586 µg/l, the amount of citrinin was well above the upper limit for beverages.

**Conclusion:**

The values obtained here emphasize that fungal contaminants and mycotoxins in bottled water should be included in the water analysis criteria. To the best of our knowledge, this is the first detailed research in this field in Turkey.

## Zusammenfassung

**Problemstellung:**

Die chemische Zusammensetzung und die mikrobielle Kontamination von kommerziell abgefülltem Wasser werden gemäß den Vorschriften des öffentlichen Gesundheitsdienstes kontrolliert, aber es gibt Lücken bzgl. des Nachweises von Pilzen. Aus diesem Grund wurden in 19-Liter-Polycarbonatflaschen abgefüllte kommerzielle Flaschenwasser-Marken aus verschiedenen Wasserquellen in Westanatolien, die in Izmir und benachbarten Provinzen verkauft werden, zufällig ausgewählt und analysiert.

**Methode:**

Das Pilzscreening wurde in Wasserproben mit Hilfe der Membranfiltrationstechnik durchgeführt. Die Anzahl wird als Koloniebildende Einheiten (KbE)/100 ml angegeben. Die isolierten Pilze wurden zunächst phänotypisch analysiert, um eine hypothetische Identifizierung zu ermöglichen. Zur genotypischen Identifizierung wurde die Internal Transcribed Spacer (ITS)-Region mittels Polymerase-Kettenreaktion (PCR) unter Verwendung von ITS1/ITS4-Primern amplifiziert. Die Sequenzierungsergebnisse wurden durch BLAST mit der NCBI-Datenbank verglichen. Für 31 Proben wurden Spezieszuordnungen vorgenommen. Es wurden 25 Schimmelpilze und 6 Hefen identifiziert. Die molekulare Identifizierung wurde durch die Aktin-Genregion unterstützt.

Das Potenzial der isolierten Penicillium-, Aspergillus-, Alternaria- und Cladosporium-Arten zur Bildung von Mykotoxinen wurde ebenfalls mittels Ammoniumdampftest, LC-MS und HPLC untersucht. Die Menge an Citrinin, dem vom *Penicillium citrinum* B4-Stamm produzierten Metaboliten, wurde mittels LC-MS mit 411,586 µg/l bestimmt,

**Ergebnisse:**

Die am häufigsten nachgewiesenen Stämme waren Cladosporium, Penicillium und Alternaria. Citrinin lag mit 411.586 µg/l deutlich über dem oberen Grenzwert für Getränke.

**Schlussfolgerung:**

Auf Grund der Ergebnisse sollte der Nachweis von Pilzen und Mykotoxinen in abgefülltem Wasser in die Kriterien für die Wasseranalyse aufgenommen werden. Soweit wir wissen, ist das die erste detaillierte Untersuchung auf diesem Gebiet in der Türkei.

## Introduction

Water demand is increasing daily because water resources remain steady despite a rapidly growing population. The United Nations aims to provide equal access to safe and accessible drinking water by 2030; globally, 2.3 billion people currently lack access to safe water [1]. Water quality is vital for humans and has a significant impact on health. Natural spring water is generally considered safer, more reliable, and cleaner than municipal water systems. Thus, there is a general trend in cities towards the consumption of bottled water [2]. However, the quality and storage period of bottled water vary depending on its source, processing, transportation, storage, treatment, bottling, and the type of packaging material used. Accordingly, packaged waters may contain many chemical and microbial contaminants. For this reason, the chemical and microbial content of water should be monitored according to universally applicable legal standards.

Bottled water and its quality standards differ from those of local tap water sources in Turkey. In addition, bottled water in the European Union countries has a lower microbial load than tap water. The “Regulation on the Production, Packaging and Sale of Natural Mineral, Drinking and Medical Waters,” published by the Turkish Ministry of Health, has enabled many companies to invest in this area and to establish standards [3], [4]. Unfortunately, some producers do not fully implement this regulation for packaged water. This regulation applies only to bacteriological analyses in Turkey, as in many European countries; however, fungal analyses are not performed.

Water and soil resources are increasingly polluted due to the excessive and uncontrolled application of chemicals used in agriculture and mineral exploration activities. As a result of such practices, the natural flora is disturbed, and the soil microbiota is destroyed, which also affects the cleanliness of water resources. Therefore, resistant harmful fungi become dominant and the mycotoxins they produce contaminate groundwater resources [5]. Fungal mycotoxins can be defined as low molecular weight compounds naturally produced as secondary metabolites by some filamentous fungi and are extremely dangerous not only for human health but also for animal health [6].They can cause various diseases, allergic reactions, and death in humans and other vertebrates, sometimes in the short term through acute illness or in the long term through extinction [7]. Given the extremely high toxicity of mycotoxins, their presence in packaged water, accumulation in the human body, and their effects on human health are unpredictable.

Today, bottled water is the primary source of drinking water and is a truly global market, even in remote areas of developing countries [8]. Measurement of microbial pathogens in bottled water is a very important issue in terms of food safety. It has been noticed that since the last century, research and analysis on food safety have focused more on pathogenic bacteria as microbial agents, but more recently, awareness of the harm resulting from fungal agents and mycotoxins has increased [9]. Many factors are important in the increased incidence of microbial agents in water, such as pollution of agricultural areas by people's wrong practices, uncontrolled urbanization and expansion of industrial areas, which trigger pollution of the environment of natural water resources. Natural water resources are becoming limited. These are attracting more attention. Unfortunately, detrimental factors that can increase the risk of water contamination are on the rise. Simultaneously, it would not be surprising to detect environmentally friendly fungi in the natural diversity of water resources; therefore, it is essential to distinguish between these and harmful fungal agents that produce mycotoxins, which threaten water safety. To this end, molecular identification of fungi isolated from water samples is necessary, and sequencing results are a valuable method for characterizing fungal agents [10].

This study aimed to investigate the fungal contaminants and toxigenic properties of bottled water sold in national markets in and around Izmir. Water from the springs in western Anatolia is sold commercially in western cities such as Izmir. Numerous studies have been conducted on the isolation and identification of fungi, the detection of their toxins, and the presence of toxigenic gene regions in bottled water in many countries. Although there are more than 250 local and foreign companies in the packaged-water sector in Turkey, there are few studies on this subject, and no detailed study on the detection of mycotoxin-producing fungi. 

## Materials and methods

### Water sampling 

This study examined various labelled water brands. Samples were collected from 19-L polycarbonate bottled water containers from nine different companies, each holding a significant market share in the city of Izmir and its surrounding areas (Turkey). The water samples from each company were coded using the initials of the brands and assigned a capital letter.

### Isolation and morphological characterization of fungi 

Approximately 100–200 ml of bottled water samples from each water brand were analyzed. Samples were passed through 0.45-µm diameter nitrocellulose sterile gridded membrane filters (Millipore S-Pak, CA, USA) under aseptic conditions. The filters were placed onto Petri plates containing dichloran rose bengal chloramphenicol agar (DRBC), which is generally used for the isolation and counting of fungi. Then, plates were incubated at 27°C for up to 7 days [11]. Growing fungal colonies in the petri dishes of each company were checked, and counting began on day 3 [12].Emerging colonies were transferred to malt extract agar (MEA), Czapeckyeast agar (CYA)and potato dextrose agar (PDA) to macroscopically and microscopically examine the resulting fungi [13]. Moulds were incubated at 27°C for 3–5 days, and yeasts at 30°C for 24–72 h [14], [15]. Microscopic examination was performed under a light microscope, on culture slides or microscope slides, and was in some cases stained with lactophenol blue or crystal violet solutions [16].

### Fungal DNA extraction 

Approximately 1 cm^2^ of mycelium growth was harvested from freshly prepared fungus cultures and transferred to sterile tubes. The mycelia were ground in the presence of sterile glass beads. Nucleospin Plant II DNA Purification Kit (MACHEREY-NAGEL, Düren, Germany) was used for the isolation of genomic DNA. The manufacturer's protocol for extracting DNA from mould and yeast was adapted according to the literature [17]. The obtained genomic DNA was stored at –20°C for later use in molecular identification. The DNA quantity, purity, and integrity were evaluated byagarose gel electrophoresis.

### PCR conditions and sequencing 

The ITS1-ITS4 region of the DNA of the isolated fungal strains was amplified by polymerase chain reaction (PCR) using the universal primer pair ITS1:5‘-ACCAACCGTGAGAAGATGAC-3’ and ITS4:5‘-TGATGGAGTTGTAGGTGGTT-3’ [18]. The PCR mix was performed in 50µl of reaction volume containing1X Taq DNA Buffer, 2 mM MgCl_2_, 0.2 mM dNTPs (Geneaid Biotech Ltd.), 0.5 µM of each primer (Sigma), 0.25 U of Taq DNA Polymerase (Gene Aid) and 40 ng of template DNA. The amplification was carried out in a programmable digital thermal cycler (Thermo Fisher Scientific, Waltham, MA, USA). The PCR reaction involved initial denaturation at 94°C for 5 minutes, followed by 40 cycles of serial denaturation at 94°C for 1 minute, primer annealing at 55°C for 2 minutes, and elongation at 72°C for 2 minutes, with a final extension step at 72°C for 7 minutes.

The DNA region encoding actin, which is also reliable in phylogenetic analyses and species identification of eukaryotes, was amplified using the primer pair; Act-1: 5‘-ACCAACCGTGAGAAGATGAC-3’ and Act-4: 5‘-TGATGGAGTTGTAGGTGGTT-3’ [19]. The PCR mix was prepared as previously described. The PCR amplification was performed following one cycle of 5 minutes at 94°C, 35cycles in three steps the first for 30 seconds at 95°C, the second for 1 minute at 55°C, and the third for 1 minute at 72°C followed by a final cycle of 7 minutes at 72°C [20].

The DNA sequence was analyzed unidirectionally for the ITS region and bidirectionally for the actin-coding region by MedSanTek Laboratories (Istanbul, Turkey) using the Sanger dideoxy sequencing method. The sequence results were evaluated using Geneious basic software 10.1 for sequence analysis and alignments. The obtained nucleotide sequences were compared with data from the National Center for Biotechnology Information (NCBI) for taxonomic identification based on sequence similarity using the Basic Local Alignment Search Tool (BLAST).

### Ammonium vapor test 

The genotypically identified strains, especially those with the potential to produce toxins, were subjected to an ammonium vapor pre-screening test formycotoxin screening. For this purpose, the identified fungal strains were grown on yeast extract sucrose agar (YES) medium enriched with MgSO_4_.The strains were inoculated as a single colony in the center of the petri dish containing the prepared medium. The cultured fungi were incubated in a dark environment at 27°C for 3–5 days. Petri dishes containing colonies growing on this medium were inverted, and petri lids were treated with 1.5 ml ammonium hydroxide. After re-incubation, whether or not they produced toxin was evaluated depending on the color change of the colony. Reddish to dark brown pigmentation of the colonies indicates mycotoxigenic potential, while no change in color indicates no mycotoxin production [21].

### Mycotoxin detection and quantification by LC-MS and HPLC 

The selected strains were inoculated into 100 ml of liquid YES medium supplemented with MgSO_4_, prepared in a buffered solution of 1M citric acid monohydrate and 1M Na_2_HPO_4_, and adjusted to pH 4.0±0.2. Submersed fermentation was performed for 7 days in a rotary shaker at 150 rpm and 25°C in the dark. The biomass was then removed by filtration through Whatman paper, and then the supernatant was passed through a 0.2 µm Millipore sterile filter [22]. Samples were stored at 4oC before sending for analysis (MedSanTek Turkey). The LC-MS (liquid chromatography-mass spectrometer) and HPLC (high-performance liquid chromatography) analyses were performed according to their laboratory methodology.

## Results

### Fungal contaminants in packaged water 

46 fungal strains were isolated from packaged water samples collected from nine companies engaged in significant commercial trade, and 32 fungal strains were identified in Izmir and the surrounding areas (Table 1 (Tab. 1)). The highest microbial load was found in the water sample from water brand “S”, followed by the “F” and “B” brands (Table 1 (Tab. 1))

The water sample from company Ba exhibited the lowest number of cfus. The fungal contaminants that emerged from the sampled water were collected from the filtration membrane incubated on DRBC for further analysis and identification. The highest number of isolates was obtained from water brand “S”, accounting for 19.6% of the fungi analyzed in this study. Only two fungal strains were isolated from water brand “Ba” (Table 1 (Tab. 1)). In contrast, 1.5-L PET bottles from water company “B” were analyzed for control purposes, but no microbial agents were detected in these plastic water bottles.

**Figure 1:**
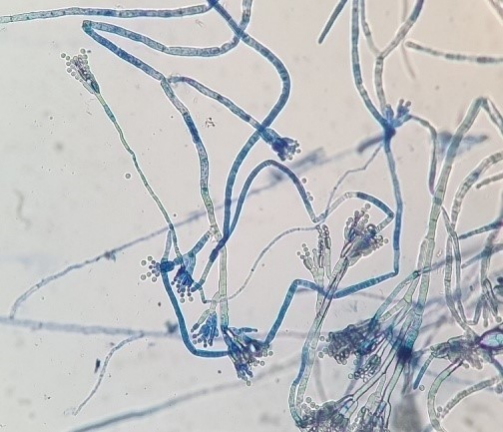
Brush-shaped conidiophores and chained conidia in *Penicillium* spp. S_3_ stained with lactophenol cotton blue (magnification 40x)

**Figure 2:**
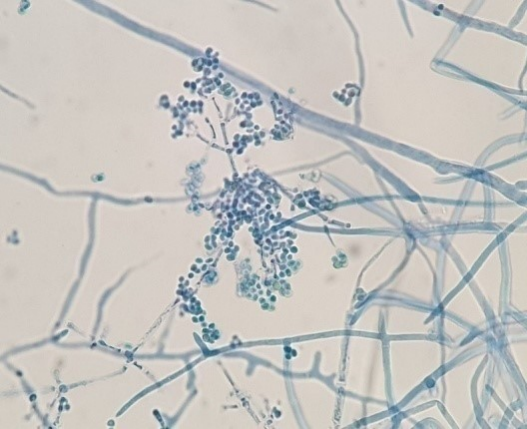
Multibranched conidiophores bearing masses of unicellular conidia in *Trichoderma* spp. Ş_4_ stained with lactophenol cotton blue (magnification 40x)

**Figure 3:**
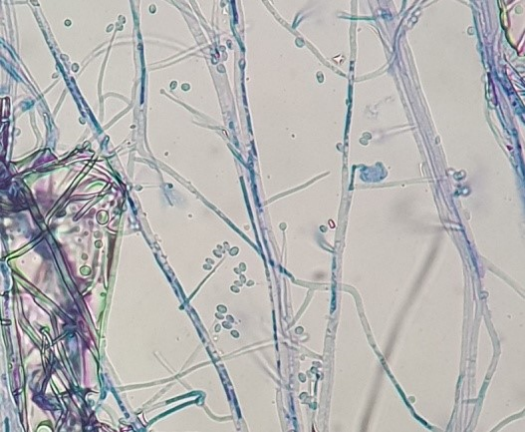
Microscopic view of *Simplicillium* spp. B_2_ stained with lactophenol cotton blue (magnification 40x)

**Figure 4:**
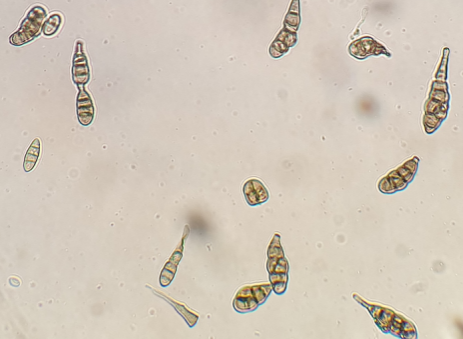
*Alternaria* spp. Ö_3_ microscopic view (magnification 40x)

**Figure 5:**
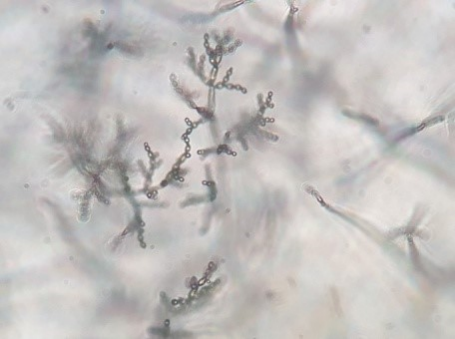
*Cladosporium* spp. F_4_ microscopic view (magnification 40x)

The purified fungal isolates were examined under a microscope on stained culture slides to reveal the growth morphology of conidiophores and their branching features, as well as the shape and arrangement of conidia, providing initial diagnostic findings. Isolates A_2_, B_3_, B_4_, S_1_, S_2_, S_3_, S_4_, S_6_, S_7_, Z_3_, and Zz_3_ exhibited *Penicillium* spp. morphology, with unicellular conidia arranged in chains on brush-shaped conidiophores, differentiated from septate hyphae (Figure 1 (Fig. 1)). *Trichoderma* spp. were identified among the isolates from Ş_4_ bottled water (Figure 2 (Fig. 2)). *Acremonium* spp. and *Simplicillium* spp. strains, coded B_5_ and B_2_, were also identified through microscopic analysis of the slide cultures (Figure 3 (Fig. 3)). Additionally, some dematiaceous fungi were isolated, including *Alternaria* spp. (Figure 4 (Fig. 4)) and *Cladosporium* spp. (Figure 5 (Fig. 5)), which were identified at the genus level after microscopic examinations. Alternaria conidia exhibited multi-celled conidia with transverse and longitudinal septa, while Cladosporium showed sympodial-branched conidiophores with chained conidia ends.

Definitive identification of all isolates was conducted through molecular analysis focusing on ITS coding regions. Among the total of 46 fungal isolates, 31 strains were identified at the genus or species level (Table 2 (Tab. 2)). By comparing the sequences of the ITS region of the studied fungal strains with the NCBI database, high identity percentages were achieved (99–100%), enabling the assignment of the strains to a putative species- or genus-level classification. 

**Figure 6:**
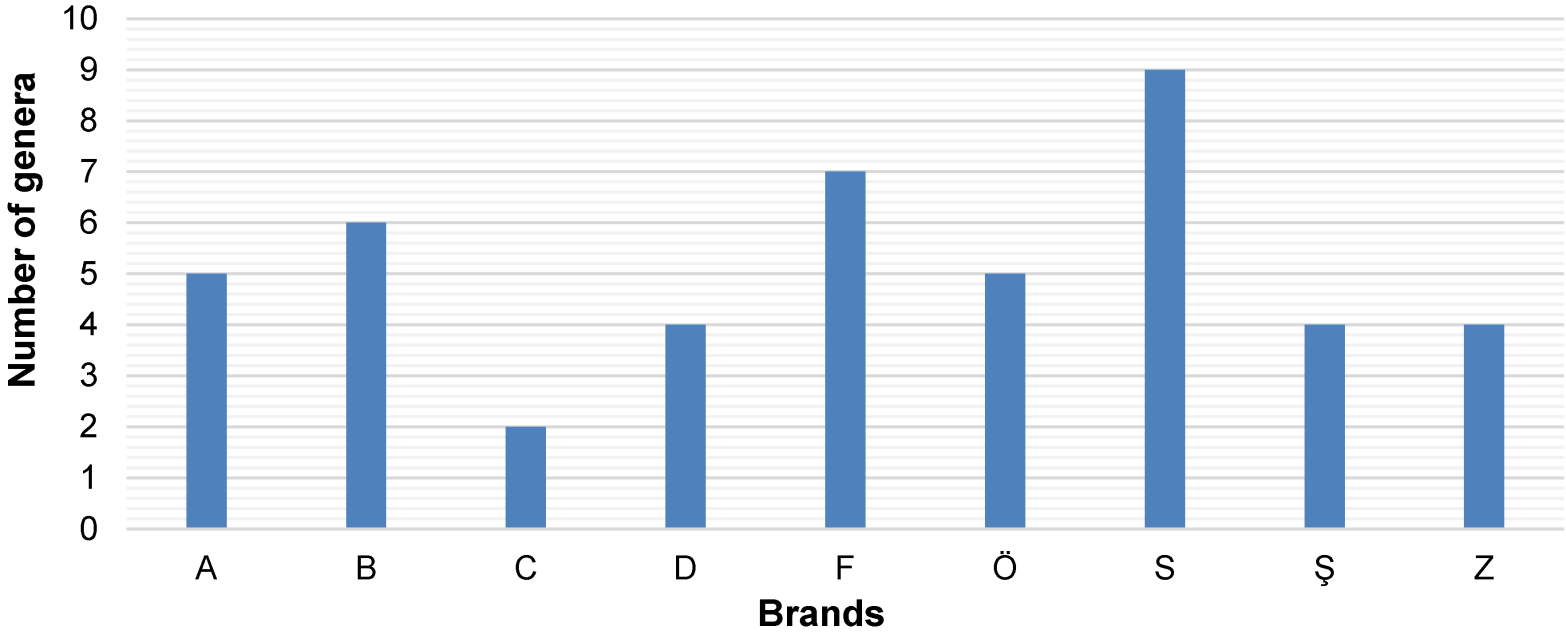
The distribution of isolated fungal strains according to the water brands. (The capital letters on the x-axis are the codes for the packaged-water companies)

**Figure 7:**
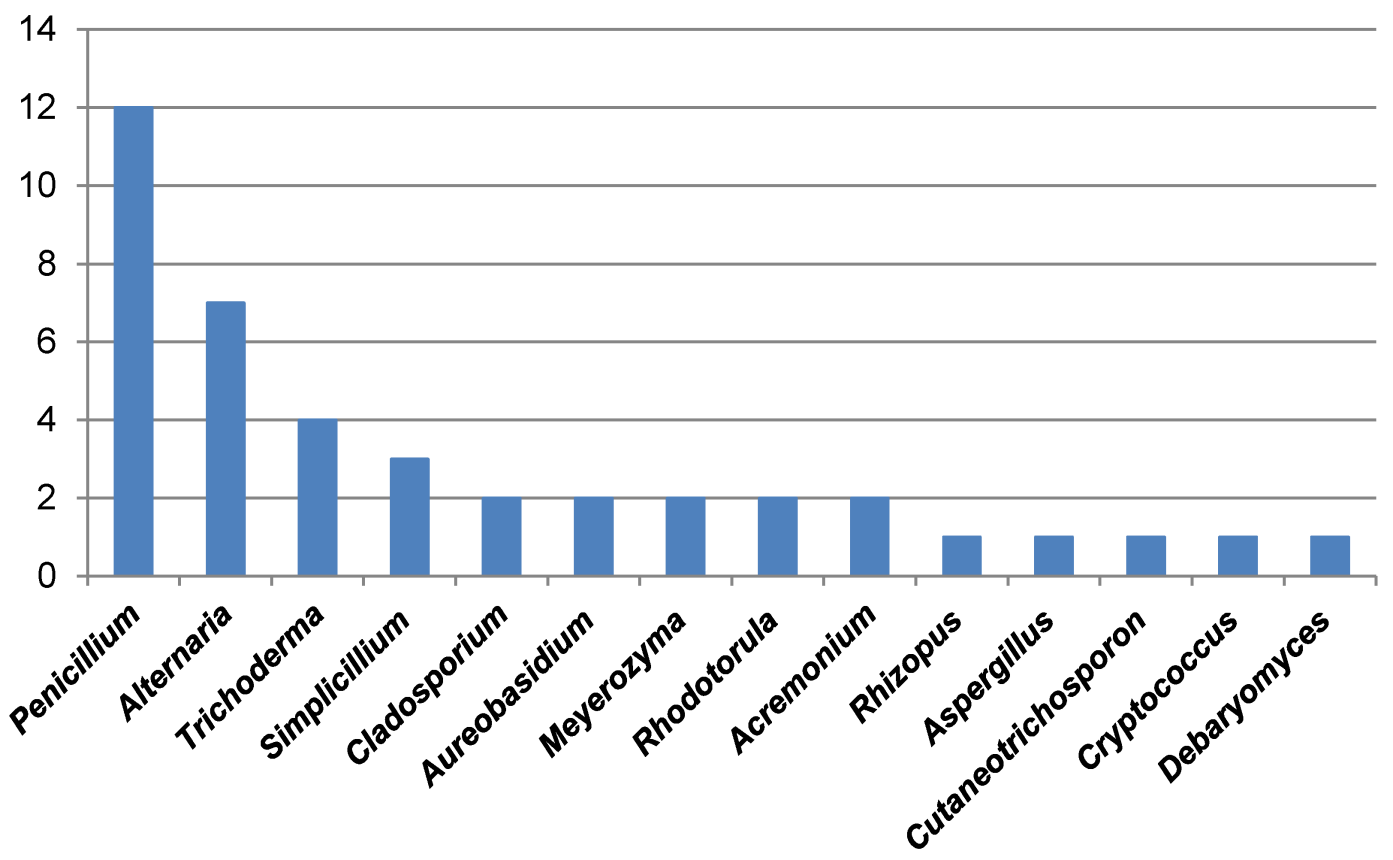
Genera and number of species per genus identified among the tested brands of bottled water

To confirm ITS identification, species-specific validation was obtained via the actin gene of the resulting PCR amplicons, indicating a size of approximately 800 bp corresponding to the relevant actin DNA regions in mould strains. Based on the identification results, the most abundant fungal contaminants in bottled water belonged to the Penicillium and Alternaria genera (Figure 6 (Fig. 6) and Figure 7 (Fig. 7)). Furthermore, fungi from the Acremonium, Cladosporium, Trichoderma, and Simplicillium genera were identified as fungal agents in various bottled water brands. Yeasts were also detected among the agents found in the analyzed water samples. Based on the identifications made in the ITS gene region, the yeast contaminants *Cutaneotrichosporon moniliforme*, *Debaryomyces hansenii*, *R. mucilaginosa*, and *M. guilliermondii* were identified at the species level.

**Figure 8:**
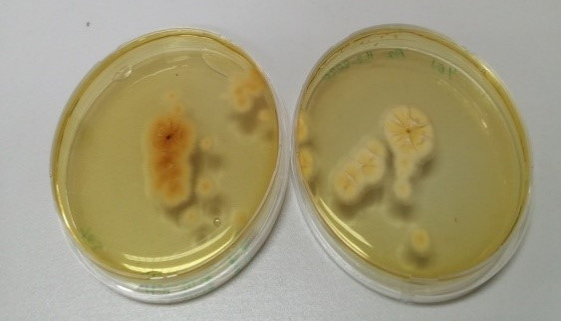
Ammonium vapor test revealing mycotoxigenic potential of strain S_1_ (right) compared to non-toxigenic fungi B_2_ (left)

The fungal strains with high toxigenic potential as given in the literature were selected from the identified strains and subjected to an ammonium vapor test (21). The samples were evaluated according to the color changes resulting from the stress condition during the vapor test. Fungal cultures in which the colony color changed to dark brown were deemed positive, indicating their potential to produce toxins Figure 8 (Fig. 8)). Screening via the ammonium vapor test revealed that nine strains were mycotoxin producers. The most intense color changes were observed in S_1_ and B_4_ isolates. 

Three mould isolates, B_2_, S_1_ and B_4_, were further chosen for HPLC and LC-MS analysis. S_1_ and B_4_ were selected due to their mycotoxin production potential as revealed by the ammonium vapor test, while B_2_ was used as control, as *Simplicillium* spp. are not known to produce mycotoxins. Toxin screening was performed by HPLC on the fermented broths of B_2_ and S_1_ strains. Patulin was found as one of the metabolites of the S_1_ strain, at a level of 9.73 ppb, while it was not detected in strain in B_2_. Additionally, citrinin was identified in the fermented broth of the *P. citrinum* (B_4_) strain at concentration of 411.586 ppb (Attachment 1).

## Discussion

Microbial contamination of water is of great importance for public health. Health risks associated with bacterial pathogens are addressed in regulations governing the use of drinking water. The effects of changing climate conditions on microbial diversity, soil and water flora are becoming increasingly polluted due to chemical pesticides, fertilizers used in agriculture, and increasing unplanned urbanization. However, the regulations about bottle water remain the same. Numerous literature reviews suggest that *Penicillium* spp. and *Alternaria* spp. are the primary contaminants in bottled water. Our study also observed that packaged waters were mainly infected with Penicillium and Alternaria strains. Yeasts such as *Debaryomyces hansenii* (also known as *Candida famata*) and *M. guilliermondii* were also reported to be found in bottled water samples [23]. In addition to these yeast strains, *R. mucilaginosa* and *Cryptococcus (C.) neoformans* strains were also detected in the present study. 

Filamentous fungi were detected in samples from all brands. The number of fungi in the samples varies between 9 and 55 cfu/100 ml. The highest number was 55 cfu/100 ml in S-coded bottled water samples, and the lowest values were 9 and 13 cfu/100 ml in Ba- and D-coded samples. Considering the number of fungi in a 100-ml sample of packaged water, there is no specific quantity limit for fungi in the regulations; the limit for non-harmful microorganisms is 20 cfu/ml, and the limit for pathogenic microorganisms is 0 cfu/100 ml (Regulation on water for human consumption [24]). The presence of non-pathogenic fungi among the fungi isolated here is a natural and expected situation. The 20 cfu/ml rate stated in the regulation is also given for this reason. However, according to the regulation, pathogenic fungi such as *Penicillium* spp. and *Alternaria* spp. were detected in most of our samples. 

To confirm the taxonomic identification at the both the genus and determine the species level, ITS1 and ITS4 universal primer pairs were used to amplify the ITS1-5.8S-ITS4 regions, after which strain identification was performed. The actin gene region also supported the identifications. To summarize, 29 isolates were identified as genus and species according to the ITS gene region, 22 of which were moulds, and the remaining 7 were yeasts.

Fungi not only cause adverse health effects on animals and humans, but also their secondary products play a role in these effects [25]. More than a hundred mycotoxins are known, and most of are synthesized by some species belonging to one of three fungal genera: *Aspergillus*, Penicillium, and Fusarium [26]. In the long term, mycotoxins can contribute to food and beverage contamination, skin irritations and allergic reactions, and may also lead to an increase in opportunistic systemic mycosis in immunocompromised patients [27]. The effects of mycotoxins, which normally show extremely high toxicity, on human health cannot be predicted due to their accumulation in the human body over time and their presence in packaged water [28]. Therefore, the identification and quantification of mycotoxin exposure are extremely important. Many screening methods have been developed to easily detect the presence of mycotoxins in samples or fungal cultures. In a study conducted to develop a simple and rapid screening method for detecting *Monascus* spp. isolates capable of producing CIT using coconut cream agar (CCA), fungi were detected based on their fluorescence upon exposure to UV light when grown using CCA [29]. Some of these methods are based on direct observation of fungal cultures in special culture media, such as the ammonium vapor test on YES agar.

Citrinin (CTN) mycotoxin was first detected in *P. citrinum*. Later, it was found that the CTN metabolite can be synthesized by *P. citreoviride, P. aurantiogriseum, P. palitans, P. purpurescens, P. expansum, P. verrucosum*, *P. citreonigrum, Aspergillus (A.) terreus, A.s candidus*, and *Monascus ruber* [30], [31]. It has been reported that citrinin can cause chronic diseases in humans and animals [32]. It is well known that long-term consumption of citrinin can cause kidney damage in humans because it inhibits water absorption in the kidneys. In addition, numerous reports have indicated that CTN exposure causes nephrotoxicity, hepatotoxicity and chromosomal abnormalities in experimental animal models [33]. However, limited evidence in animals has categorized it as unclassifiable for human carcinogenicity by the International Agency for Research on Cancer in group 3 [34].

In our study, *P. citrinum* and *P. chrysogenum* were found positive for toxin production among all isolates screened by the ammonium vapor test, and the amount and type of toxin in the metabolite they produced were determined. The amount of citrinin synthesized by *P.citrinum* (B_4_) was determined as 411.586 µg/kg. According to the EU Commission Regulation, there is no threshold value for packaged water. Limits are determined only for packaged beverages such as fruit juice, wine, beer, and soda. Therefore, the amount and type of toxin we obtained were evaluated according to the limits for these beverages. The EU Commission [35] determined the ochratoxin limit as 5 and 10 µg/kg for processed and unprocessed foods and 2 µg/kg for fruit juice and wine. However, the Turkish Food Codex Pollutants Regulation of the Ministry of Food and Agriculture does not include a toxin limit for packaged water [36]. In this regulation, the ochratoxin limit for wine and fruit juice is 2 µg/kg, and the upper level of patulin for fruit juice and alcoholic beverages is 50.0 µg/kg. Notably, in this study, the amount of citrinin produced by the B_4_ strain affected the upper limit of total mycotoxins in wine and fruit juice determined by two regulatory agencies.

Patulin (PAT) is a polyketide lactone mycotoxin produced by many species of Penicillium, Aspergillus and Byssochlamys [37]. *P. expansum*, for instance, is a significant producer of PAT, associated with serious health problems and posing an economic burden. Studies have shown that PAT affects kidney cells and has neurotoxic, immunotoxic, mutagenic and carcinogenic properties [38]. Due to its toxicity and adverse health effects, the maximum tolerable daily intake of PAT for humans has been tentatively determined as 0.4 µg/kg body weight [39]. European Commission Regulation (EC) No. 1881/2006 sets the maximum permissible levels for PAT in various dietary products [40]. The regulation states that the maximum acceptable level of PAT should not exceed 50 µg/l in fruit juices, alcoholic beverages and cider, 25 µg/kg in solid apple products and 10 µg/kg in foods for infants and young children [40]. In the present study, the quantity of PAT obtained in strain S_1_ was 9.76 µg/kg, which is below the upper limit but very close to the critical range for children. Therefore, PAT may be a potential health risk factor.

The results of the current research show that some fungal species found in commercially available packaged water clearly produce toxins. The presence and identification of fungi in such water have been debated and associated with organoleptic defects related to taste, odor, and especially allergic reactions [41], [42]. Therefore, the toxin-producing potential of these fungi makes our study important for human health. The literature review stated that *Penicillium* spp. were commonly identified in drinking water, and some of these species could synthesize patulin and citrinin metabolites. Apparently, some studies on water exist in the literature, but studies on toxin-producing fungi in packaged water are few. Many physical factors, such as storage time, ambient temperature, pH, oxygen water activity, and nutrient composition, can trigger fungal growth. Also, these environmental factors can affect the PAT biosynthesis mechanism in many filamentous fungi [43]. When a fungus finds suitable conditions, it starts to multiply and secrete mycotoxins, but the main reason for mycotoxin production is still unclear today [44].

The chemical composition of bottled water varies among different brands. Moulds are microorganisms with complex enzyme systems and can thrive even in very nutrient-poor environments, e.g., under low concentrations of Ca, Mg, K, S, Fe, and Mn [45]. The conductivity measurement in the chemical analysis table of packaged water indicates the presence of metal ions; higher concentrations of metals and salts such as CaCO_3_ and Mg result in increased conductivity. Water conductivity reflects the amount of dissolved minerals (ion content) such as Ca, Mg, Cl, SO_4_, PO_4_, and HCO_3_ [46]. In the present study, different yeast strains were observed in different brands: *Debaryomyces hansenii* (nitrite assimilation) in brand F,* C. neoformans* (capsule in low Fe medium) in brand A, *M. guilliemondii* in brand C, *Cryptococcus albidus* in brand H, and *R. mucilaginosa* (nitrogenous compounds) in brand G, particularly in bottled water brands with the highest conductivity levels. This result suggests that the interaction between mineral content and the plastic of bottled water in addition to microplastics may promote the growth of yeast strains. This indicates that the chemical composition of these brands of water should also be analyzed.

Polycyclic aromatic hydrocarbons (PAH), acrylamide, benzo(a)pyrene, ammonium, mercury, nitrate chloride, sulphate, copper, iron, and manganese levels in packaged waters increase based on environmental factors such as heat and light. In some brands, these levels exceed the guidelines set by TSE, the World Health Organization (WHO), the U.S. Environmental Protection Agency (EPA), and EU standards [47], [48], [49]. This can alter the presence of pathogenic fungi in the water and their potential to produce mycotoxins. For instance, the expiration date for cosmetic products, such as creams and makeups, is indicated on the packaging from the moment it is opened, in accordance with the cosmetics legislation of the Turkish Ministry of Health [50]. Similarly, the safe usage period (starting with opening the cap) of bottled water should be indicated on the package.

The European Union Drinking Water Directive specifies parameter values for bacteria but not for fungi [51]. Aside from some toxin limits in the EU Commission Regulation [49] mentioned earlier, only a limited number of member states have additional, more specific regulations. The Czech Republic and Hungary have adopted a similar approach in their drinking water legislation, requiring microscopic examination of drinking water and setting separate parameter values for groups of organisms. Hungary applies a parameter value of 0 individuals/L for fungi [52], [53]. Furthermore, Swedish legislation is the only legislation in Europe that requires direct detection of fungi by culture (limit: 100 cfu/100 ml) [54]. Clearly, to guarantee the health of drinking water, it is essential to reduce the differences between limits and universalize the regulations. One study has shown that most brands of water sold even in New Zealand do not meet drinking water standards [55]. This indicates that microbiological quality criteria must be reviewed to ensure that water worldwide is safe and acceptable. A globalized and standardized strategy for monitoring exogenous hazardous substances, such as fungal mycotoxins in drinking water, should be developed for human health safety.

Recent developments suggest that freshwater resources are gradually diminishing worldwide due to the combined effects of severe human activities and climate change. Thus, it is necessary to reconsider both the management of water resources and the standards that define water quality. Natural waters are seldom polluted by the physical and chemical decomposition of surrounding rocks. Additionally, soil pollution arises from the expansion of agricultural areas near water resources, the use of pesticides and fertilizers, and the accumulation of industrial waste and mineral deposits. Consequently, pollution in the soil also contaminates groundwater. The chemical composition of surface and groundwater changes upon interaction with geological units and soil pollutants. These changes can result in increased concentrations of specific ions in natural waters. These waters may also contain heavy metals that can adversely affect human health, and the chemical properties of water also influence the presence of fungi in water systems. Another contributing factor is that the risk of contamination may rise seasonally due to decreased rainfall. Fungal load can be assessed by analysing water samples taken during the summer months when water consumption is high, as well as examining fungal load in samples collected during the rainy season. Generally, it has been demonstrated that rocks with an alkaline pH are more susceptible to fungal colonization than those with an acidic pH. In our study, most bottled waters were found to be alkaline. Therefore, bottled water labels in Turkey should be updated, particularly to include the results of the primary ion analysis.

It is evident that only a few countries in the world, including the Czech Republic, Hungary, and Sweden, have regulations that mandate the direct detection of fungi through culture. There is no information available on the mycotoxin content in packaged water, nor is there guidance on the permissible limits for this class of compounds. Currently, only beverages like fruit juices are under investigation. Estimating mycotoxin levels is stressed as a necessary component of the microbiological analysis of water samples. The existing microbial quality standard, which is part of the food standards code, should be updated to include mineral water, bottled water, and packaged ice. To prevent microbial health issues related to drinking water, it is crucial to periodically check key points of water sources during handling, transportation, bottling, storage, and delivery to the end consumer, ensuring compliance with quality standards.

Fungal and mycotoxin contamination in packaged water can occur at every stage of the process, from filling at the plant to storage and packaging. Additionally, while spring water may be clean at the source, the use of bottles made from reusable materials may lead to contamination. As noted, mycotoxin production is influenced by several factors, including environmental conditions, the use of pesticides, fungicides, and fertilizers, as well as strain specificity, variation, and interactions among toxigenic fungi species.

### Limitations

Due to the limited resources available, this study could not identify all potential pathogens that may contaminate water, including other pathogenic bacteria, viruses, and parasites, although they can also occur [56].

## Conclusions

This study offers an updated perspective on the presence of filamentous fungi in commercial bottled water. Certain shortcomings and disadvantages were evident from the outset, prompting suggestions based on the needs identified in our research. Mycotoxins such as citrinin, patulin, and ochratoxin produced by fungal strains warrant detailed investigation, and a rapid method for detecting toxigenic fungi should be developed using HPLC and LC-MS with the necessary validation studies.

## Notes

### Competing interests

The authors declare that they have no competing interests.

### Funding

None. 

### Acknowledgements

This project is a PhD thesis carried out in the laboratories of Ege University’s Department of Biology and Division of Basic and Industrial Microbiology. We would like to sincerely thank Prof. Dr. Mustafa Ates, the head of the department and the academicians of the Division of Microbiology. 

### Authors’ ORCIDs

- Tunç G: https://orcid.org/0009-0006-9812-1784
- Uçar F: https://orcid.org/0000-0002-8140-7448
- Telli O: https://orcid.org/0000-0001-7337-8109
- Boiu-Sicuia OA: https://orcid.org/0000-0001-7830-4109
- Lima N: https://orcid.org/0000-0003-2185-0613

## Supplementary Material

Supplementary data

## Figures and Tables

**Table 1:**
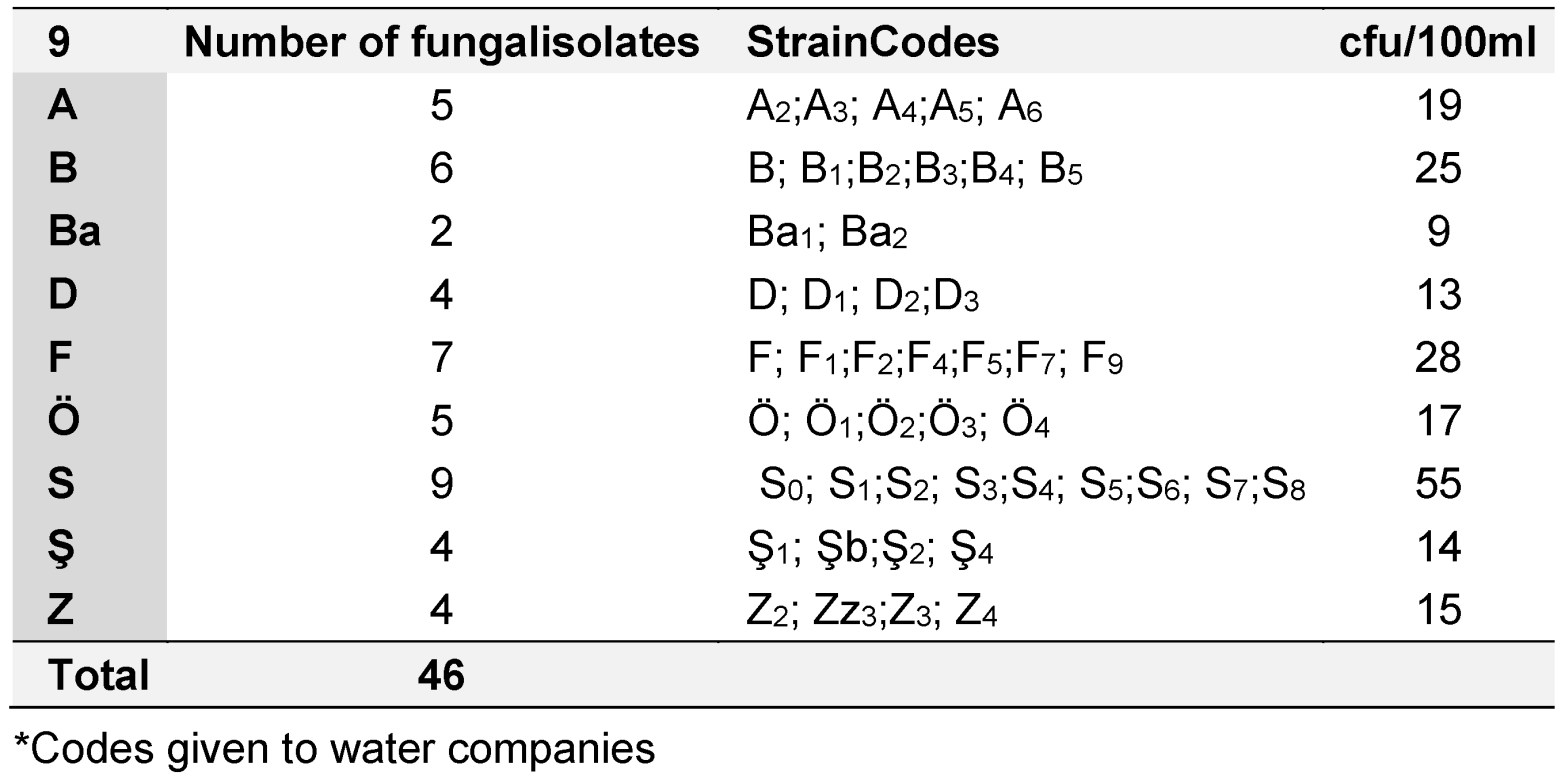
Number of fungal strains isolated from samples of bottled water

**Table 2:**
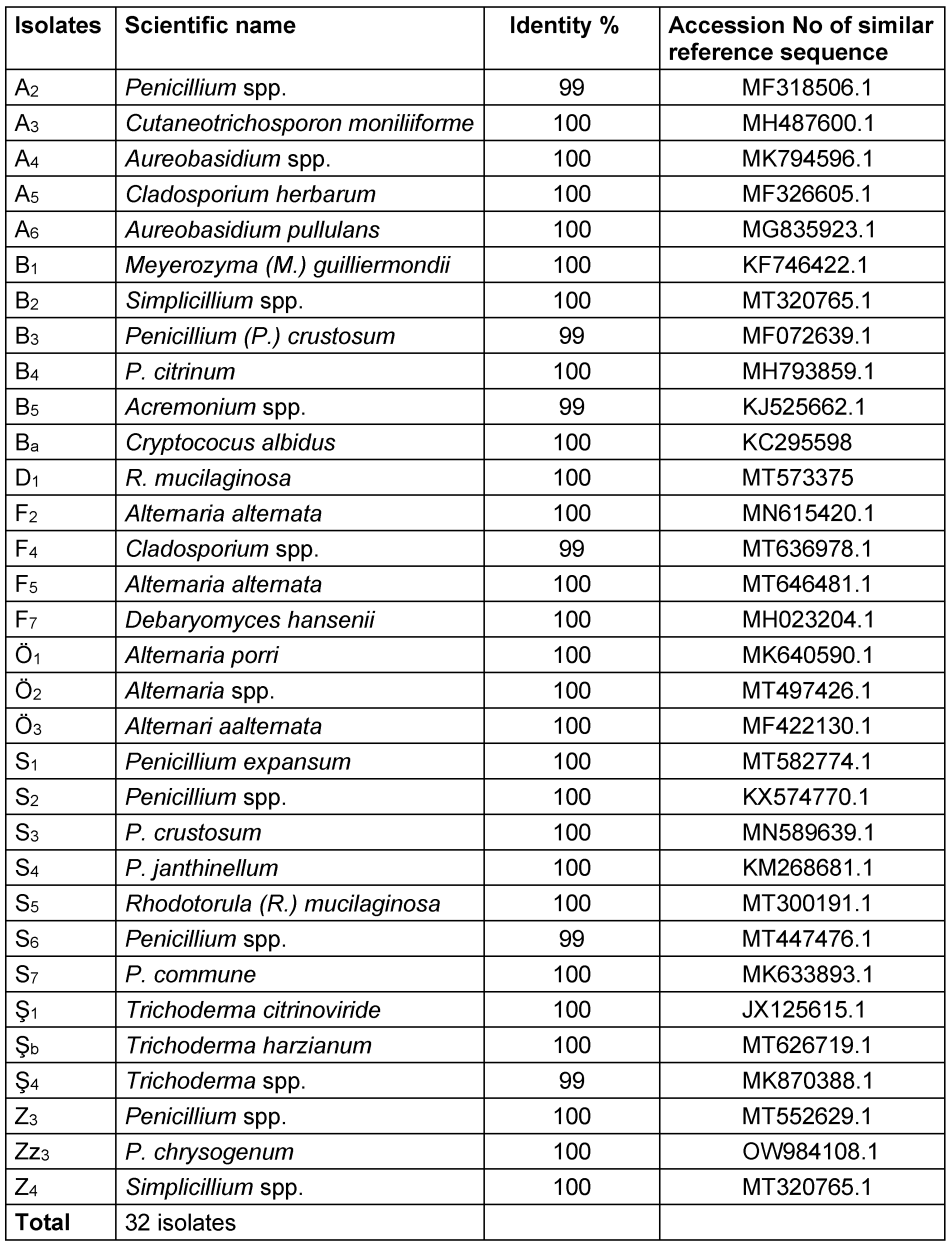
Molecular-based identification of fungi according to ITS
