# Supplementary material for: Fungal contaminants in Turkish bottled water and their mycotoxin-producing potential
Source: GMS Hyg Infect Control. 2025 Jul 14;20:Doc41. doi: 10.3205/dgkh000570 (PMC12382378; doi:10.3205/dgkh000570)
Supplement: Supplementary data [file HIC-20-41-s-001.pdf]

Attachment 1

Supplementary data

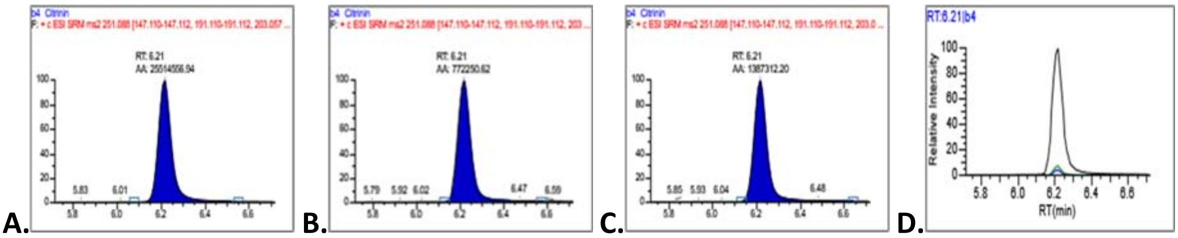

Figure 1: LC-MS chromatograms of mycotoxin produced by *Penicillium citrinum* B_4 strain in YES broth supplemented with $MgSO_4$. Images show the results obtained in 3 replicates (A, B, C) and the calculated average of these values (D).

Table 1: Mycotoxin quantification by LC-MS for *Penicillium citrinum* B_4 strain

**Toxin**	RT (min)	Ion	Ratio	Amount(µg/kg)
Citrinin	6.21<br>6.21	m/z 233.111<br>m/z 147.111<br>m/z 191.111	100.00<br>3.03<br>5.44	411.586

Attachment to: Tunç G, Uçar F, Telli O, Lima N, Boiu-Sicuia OA. Fungal contaminants in Turkish bottled water and their mycotoxin-producing potential. GMS Hyg Infect Control. 2025;20:Doc41. DOI: https://dx.doi.org/10.3205/dgkh000570
